# Dental Implantology in Acromegaly: Pathophysiological Challenges, Biomaterial Interactions, and Future Directions—A Narrative Review

**DOI:** 10.3390/jfb16110411

**Published:** 2025-11-05

**Authors:** Beata Wiśniewska, Sandra Spychała, Kosma Piekarski, Ewelina Golusińska-Kardach, Maria Stelmachowska-Banaś, Marzena Wyganowska

**Affiliations:** 1 Department of Oral Surgery, Periodontology and Oral Mucosa Diseases, Poznan University of Medical Sciences, 10 Fredry Street, 61-701 Poznan, Poland; 2 Arbor Vitae Society of Ethics in Medicine, 103 Marymoncka Street, 01-813 Warsaw, Poland; 3 Department of Endocrinology, The Centre of Postgraduate Medical Education, 99 Marymoncka Street, 01-813 Warsaw, Poland

**Keywords:** acromegaly, GH, IGF-1, bone microstructure, osseointegration, soft tissue, dental implants, biomaterials

## Abstract

Introduction: Acromegaly is a chronic endocrine disorder caused by excessive secretion of growth hormone (GH) and insulin-like growth factor 1 (IGF-1). Acromegaly leads to a wide range of systemic alterations, including metabolic disturbances, abnormalities in bone microarchitecture, soft tissue overgrowth, and morphological changes in the maxilla and mandible. All these factors may significantly complicate the planning and success of implant therapy. Study Aim: This narrative review aimed to critically analyze the impact of acromegaly on bone healing and osseointegration, with particular emphasis on the stability of implant biomaterials, and to assess whether the disease constitutes a contraindication to implant prosthetic treatment. Methods: A narrative literature review was conducted using the PubMed, Scopus, and Web of Science databases, covering publications from 2000 to August 2025. Manual screening of reference lists from key articles was also performed. Peer-reviewed publications in English, including experimental and preclinical studies, case reports, biomaterials research, and conceptual reviews, were included based on their relevance to acromegaly, bone metabolism, stomatognathic alterations, and implant therapy outcomes. No formal inclusion or exclusion criteria were applied, and methodological quality was not formally assessed, reflecting the exploratory and conceptual nature of this review. Results: Patients with acromegaly exhibit persistent structural bone deficits, such as reduced trabecular number, irregular trabecular distribution, and increased cortical porosity, despite normal or even elevated bone mineral density. In parallel, profound changes in soft tissues and dentition are observed, including macroglossia, diastemas, gingival overgrowth, and mandibular prognathism, which further complicate prosthetic rehabilitation. Animal studies suggest that GH and IGF-1 may support early osseointegration, although the long-term effects of their excess remain inconclusive. Clinical data, although limited, indicate that implant placement in patients with acromegaly is feasible when treatment is meticulously planned and carried out within an interdisciplinary setting. Standard biomaterials, such as titanium and its alloys, may undergo degradation under conditions of chronic inflammation and oxidative stress, underscoring the need for innovative solutions integrating bioactive and immunomodulatory materials, as well as patient-specific implants manufactured using 3D printing technologies. Conclusions: Acromegaly should not be regarded as an absolute contraindication to implant therapy; however, the current evidence is limited. Implant placement requires individualized planning, endocrine control, and interdisciplinary coordination. Further clinical and preclinical studies are needed to establish reliable treatment protocols for this population.

## 1. Introduction

Acromegaly is a chronic endocrine disorder resulting from excessive secretion of growth hormone (GH), which is most commonly caused by a pituitary adenoma. The disease is characterized by elevated circulating levels of insulin-like growth factor 1 (IGF-1), which mediates a broad spectrum of systemic alterations affecting both soft tissues and skeletal structures. These changes significantly influence overall systemic function as well as therapeutic potential, including implant-related treatment. Importantly, despite effective hormonal therapy, many of these alterations remain irreversible, and bone metabolism disturbances may persist for many years [1].

Within the skeletal system, acromegaly leads to profound disturbances of trabecular bone microarchitecture and, according to more recent findings, of cortical bone. These changes are driven by accelerated bone turnover stimulated by GH and IGF-1 excess. High-resolution peripheral quantitative computed tomography (HR-pQCT) studies have demonstrated microarchitectural impairments, including a reduced trabecular number, increased trabecular separation, greater heterogeneity of distribution, and elevated cortical porosity. Notably, areal bone mineral density (aBMD) is often normal or elevated, which may mask the true fracture risk [1,2].

The literature indicates that bone microarchitectural alterations revealed using HR-pQCT not only persist during the active phase of acromegaly but also following successful treatment, as confirmed in cohorts of patients in biochemical remission. Despite partial increases in volumetric bone mineral density (vBMD), structural deficits, such as reduced trabecular number, irregular distribution, and increased cortical porosity, remain present in patients in remission when compared with healthy controls, suggesting the potentially permanent nature of these abnormalities [3].

Craniofacial manifestations of acromegaly include enlargement of the mandible and maxilla, macroglossia, diastemas, altered occlusal profile, and thickening of soft tissues. These changes affect not only facial esthetics and masticatory function but also the biomechanics of the stomatognathic system, which is of critical relevance for prosthetic and implant treatment planning [4]. Malocclusion, altered muscle tension dynamics, and frequently coexisting periodontal inflammation may considerably hinder osseointegration and compromise long-term implant stability.

Despite these significant clinical and metabolic challenges, the number of patients with systemic conditions—including endocrine disorders—undergoing implant therapy has been steadily increasing. Advances in biomaterials, surgical techniques, and prosthetic planning have expanded the indications for implant placement to more complex systemic populations.

However, in the context of acromegaly, the availability of clinical data remains extremely limited. To date, the literature reports only a single case of comprehensive implant prosthetic rehabilitation in a patient with untreated, active acromegaly. A four-year follow-up assessment demonstrated successful integration of mandibular and maxillary implants without clinical or radiological evidence of failure. The authors highlighted the necessity of meticulous treatment planning, considering bone morphological abnormalities, altered occlusal relationships, and potential occlusal overload, despite the ultimately favorable clinical outcome [5]. Another isolated case describes successful bone augmentation procedures in a patient with acromegaly. Schiller et al. (2020) reported a maxillary alveolar ridge augmentation using a sinus lift technique combined with a bone substitute, with a favorable clinical result [6]. However, the authors did not provide information regarding the patient’s metabolic status, disease management, or laboratory findings, making it impossible to determine whether the procedure was performed during the active or stable phase of the disease. Nevertheless, this case suggests that reconstructive procedures may be technically feasible even in patients with endocrine-related disturbances [6].

In light of the increasing demand for implant therapy among patients with systemic disorders, an important question arises as to whether current material solutions—such as titanium implants, titanium–zirconium alloys, ceramics, or bioactive surface modifications—are sufficient to ensure durable osseointegration under the altered bone physiology characteristic of acromegaly.

The aim of this review was to provide a critical analysis of the available evidence regarding the integration of implant biomaterials in patients with acromegaly, with particular focus on bone microstructure, tissue responses, and functional performance of materials under conditions of disrupted endocrine homeostasis. This review also seeks to determine whether acromegaly should be regarded as an absolute contraindication to implant therapy, or rather as a modifiable risk factor that can be effectively managed through thoughtful material selection, surgical planning, and close interdisciplinary collaboration between dental specialists and endocrinologists.

## 2. Materials and Methods

This study was designed as a narrative literature review aimed at integrating clinical, experimental, and biomaterial-related data in the context of dental implantology in patients with acromegaly. This approach resulted from the limited availability of primary data, the rarity of the disease, and the hypothetical and translational nature of the issues addressed. The objective of the review was not to assess the efficacy of a single specific intervention, but rather to present a complex, multi-stage research strategy for future experimental and clinical studies.

The literature search was conducted using the PubMed, Scopus, and Web of Science databases, covering the period from January 2000 to August 2025. The search process was carried out between June and September 2025. The search strategy incorporated a set of keywords related to hormonal bone disorders and dental implantology, such as “acromegaly”, “growth hormone excess”, “osseointegration”, “biocompatible materials”, “dental implant”, “animal model” and others, which were adapted to each database. In total, 666 records were retrieved and imported for analysis. After duplicate removal, 526 unique publications remained. Following manual screening of titles, abstracts, and full texts, 77 studies were ultimately included in the review.

Additionally, manual citation searches and screening of reference lists from key review studies were performed. Peer-reviewed publications in English were included, including “ahead of print” articles and preprints from reputable scientific journals. The included materials comprised original research articles, case reports, in vitro and in vivo studies, conceptual papers, and narrative reviews. The inclusion was based on thematic relevance to acromegaly, bone metabolism, biomaterial performance, and implant-related outcomes.

Due to the narrative nature of the review, no formal inclusion and exclusion criteria were applied in the sense of systematic reviews. The selection of material was based on conceptual relevance and the potential usefulness for forming a coherent research strategy. The review included publications describing clinical cases of implant placement in patients with acromegaly, experimental in vitro and in vivo studies involving GH or IGF-1, and works on bone biology and skeletal metabolism under hormonal disturbances, as well as studies on bioactive implant materials, computational models, and remodeling biomarkers.

Since the aim of this study was not a quantitative analysis of intervention efficacy but rather a conceptual and translational synthesis of the available data, no formal methodological quality assessment was conducted, nor was a PRISMA diagram applied. The inclusion of studies with a low level of evidence, such as case reports, small animal experiments, or in vitro studies, resulted from a deliberate research decision adapted to the constraints of the topic.

## 3. Pathophysiology of Bone in Acromegaly

### 3.1. Bone Microarchitectural Alterations

In the course of acromegaly, chronic GH and IGF-1 excess lead to unfavorable changes in bone microstructure, despite the frequent presence of increased bone mass. In the study by Duan et al. (2021), using HR-pQCT, patients with both active disease and those after treatment exhibited reduced trabecular number, greater trabecular separation, and increased cortical porosity [1]. Although vBMD parameters partially improved following treatment, they ultimately failed to reach values observed in healthy individuals. These findings suggest the presence of persistent microarchitectural abnormalities that may not be fully captured by standard BMD measurements [1].

Furthermore, cohort analyses indicate that bone microarchitectural deficits may persist even after hormonal control has been achieved. These long-lasting alterations include reduced trabecular number, increased trabecular separation, and elevated cortical porosity. Such abnormalities may represent a permanent consequence of prolonged GH/IGF-1 overexposure and disrupted bone remodeling, persisting despite normalization of circulating hormone levels [3].

**Figure 1:**
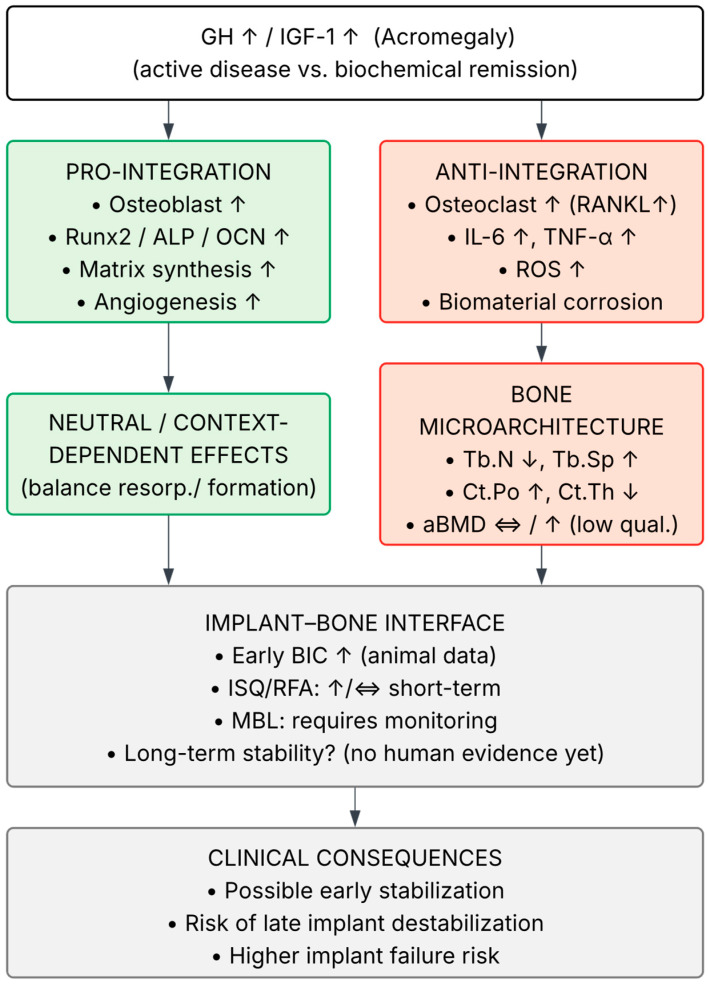
Proposed pathways of GH/IGF-1 excess in acromegaly influencing bone remodeling and implant osseointegration. Green boxes: Pro-integration pathways (osteoblast activation, matrix synthesis, and angiogenesis). Red boxes: Anti-integration pathways (osteoclast activation, pro-inflammatory cytokines, oxidative stress, and biomaterial degradation). Gray boxes: Neutral or context-dependent effects (depending on hormonal control and inflammatory status). Symbols: ↑ increase; ↓ decrease; and ⇔ balance/no significant change. Abbreviations: BIC = bone-to-implant contact; ISQ/RFA = implant stability quotient/resonance frequency analysis; MBL = marginal bone loss; Tb.N = trabecular number; Tb.Sp = trabecular separation; Ct.Po = cortical porosity; Ct.Th = cortical thickness; and aBMD = areal bone mineral density.

The mechanisms underlying these pathological changes can be explained by the complex effects of GH and IGF-1 on the balance between osteoblast and osteoclast activity. GH stimulates osteoblast differentiation while simultaneously enhancing osteoclastogenesis, which occurs partly indirectly through increased Receptor Activator for Nuclear Factor κ B Ligand (RANK-L) expression. Locally produced IGF-1 within bone acts as a critical paracrine factor regulating bone formation and resorption, independently of circulating IGF-1 concentrations [7,8]. This dual regulatory mechanism is illustrated in Figure 1, which outlines the opposing pro- and anti-integration effects of GH and IGF-1 on bone remodeling dynamics.

Clinical data, such as reduced trabecular bone score (TBS) in patients with acromegaly, including some with controlled disease, further support the notion that bone microarchitectural quality may be significantly impaired despite preserved or elevated bone mass. TBS provides a more accurate reflection of trabecular bone quality compared with conventional densitometry; therefore, TBS holds greater value for fracture risk assessment. Importantly, vertebral fractures have been documented in some patients with acromegaly despite normal aBMD values, underscoring the limitations of standard densitometry in accurately assessing true fracture risk in this population [9]. These paradoxical findings correspond to the imbalanced remodeling shown in Figure 1, where increased bone turnover under GH/IGF-1 excess leads to structural fragility despite preserved bone mass.

A summary of the reported microarchitectural changes in acromegalic bone is presented in Table 1.

Collectively, these microarchitectural alterations render bone in patients with acromegaly a biologically suboptimal environment for implant therapy. The loss of functional load-bearing microstructure compromises the ability of bone to provide mechanical support for implants, reduces osseointegration stability, and increases the risk of implant failure in this patient population. These microarchitectural and remodeling abnormalities form the biological foundation for understanding the altered interaction between bone tissue and implant materials, which is discussed in Section 4

The bidirectional role of GH/IGF-1 excess on bone remodeling and implant integration is summarized in Figure 1.

Chronic GH/IGF-1 excess in acromegaly increases bone turnover and induces persistent microarchitectural deficits—reduced trabecular number, increased trabecular separation, and elevated cortical porosity—often despite normal or elevated aBMD. These alterations help explain the paradox of preserved bone mass with impaired quality and higher fracture risk, and may compromise the mechanical environment required for reliable implant anchorage. Current evidence is largely derived from small cross-sectional cohorts and heterogeneous imaging endpoints, underlining the need for longitudinal studies integrating HR-pQCT and TBS with clinical outcomes.

The following Section 3.2 expands on how these skeletal alterations translate into maxillofacial morphological changes that further influence implant placement and functional stability.

### 3.2. Alterations of the Maxilla and Mandible

In acromegaly, patients frequently present with characteristic craniofacial changes—most notably macroglossia, mandibular prognathism, and pronounced maxillary prognathism. These features have been recognized as key diagnostic indicators since the earliest descriptions of the disease [10]. The majority of patients with acromegaly exhibit craniofacial manifestations, most commonly diastemas, macroglossia, and mandibular prognathism, with their overall prevalence estimated at nearly 80% [4].

The most common skeletal and dental manifestations of acromegaly, together with their clinical implications, are summarized in Table 2.

Macroglossia is one of the most frequently described oral manifestations of acromegaly and poses a major clinical challenge, leading not only to disturbances in mastication, speech, and breathing but also significantly affecting anatomical and functional conditions. An enlarged tongue may hinder accurate diagnostic procedures such as intraoral scanning or radiographic imaging, as well as limit surgical access during implant placement. In addition, increased tongue mass alters force distribution within the dental arches and may contribute to prosthetic instability, particularly in the posterior segments. These factors necessitate highly individualized treatment planning, consideration of altered biomechanics, and, in many cases, the use of hybrid or modified prosthetic designs to ensure functional and long-term implant success [12].

In a study by Bruno et al. (2025), based on questionnaires assessing oral health and quality of life, patients with acromegaly identified mandibular overgrowth as one of the most noticeable and burdensome symptoms [13]. The morphological basis of this condition is primarily attributed to excessive elongation and thickening of the mandibular condylar process, as confirmed via cephalometric studies in acromegalic populations [4].

Skeletal changes in acromegaly, such as overgrowth of the mandibular condyle and body, lead to anterior displacement of the mandible relative to the maxilla, resulting in a Class III malocclusion. This anatomical relationship adversely affects occlusion, facial esthetics, and masticatory function, and often justifies the need for surgical orthodontic intervention. Contemporary therapeutic approaches emphasize that patients with acromegaly may require orthognathic surgery to correct prognathism and restore proper maxillomandibular relationships [11].

Importantly, alveolar process overgrowth and changes in maxillomandibular bone structure may significantly alter anatomical conditions for implant therapy. Such cases often require modifications in treatment planning, including adjustments in implant angulation, the use of longer implants, or advanced augmentation techniques. This highlights the necessity of interdisciplinary collaboration between the implantologist, maxillofacial surgeon, and endocrinologist. The importance of such an approach is also underscored in studies analyzing the dental needs of patients with acromegaly [13,14].

Craniofacial manifestations of acromegaly—including mandibular prognathism, macroglossia, and diastemas—modify occlusion, load distribution, and surgical access, thereby complicating implant planning and prosthetic stability. These changes often necessitate individualized implant positioning, bone augmentation, and, in selected cases, orthognathic correction within an interdisciplinary framework. Evidence remains predominantly descriptive, and prospective data linking specific craniofacial patterns to implant outcomes are lacking.

Building on these structural insights, the next Section 3.3 examines how endocrine and inflammatory mechanisms—particularly GH/IGF-1-driven angiogenesis and wound-healing dynamics—may further impact peri-implant bone regeneration.

### 3.3. Inflammation, Angiogenesis, and Wound Healing

In acromegaly, chronic GH excess and elevated IGF-1 levels may influence local inflammatory processes, angiogenesis, and bone regeneration, which are key elements for successful osseointegration. Although these mechanisms have not been directly studied in the context of implantology in acromegalic patients, preclinical data suggest that IGF-1 may modulate inflammatory and reparative processes, including mesenchymal cell differentiation, macrophage recruitment, and neovascularization (Zhang X et al., 2020) [15]. However, these observations require further translational research [14,15].

While direct investigations into angiogenesis at implant sites in acromegaly are scarce, it is well established that GH and IGF-1 play critical roles in stimulating endothelial cells, thereby promoting new vessel formation [15,16]. Nevertheless, under pathological conditions, excessive vascular stimulation may result in structurally inferior vessels, thereby impairing microcirculation within healing tissues.

In the context of wound healing and implant osseointegration, the early postoperative inflammatory response plays a pivotal role. As highlighted in the review by Yi et al. (2022), hormones such as GH and IGF-1 may facilitate reparative processes by modulating inflammation and promoting angiogenesis, which could favorably impact the early phases of implant integration [17]. However, the authors stress that excessive or dysregulated activity of these hormones—particularly under endocrine disturbances—may disrupt the balance between the inflammatory and proliferative phases, potentially influencing long-term osseointegration stability [17].

An unstable inflammatory response, inadequate angiogenesis, and disturbances in the sequential phases of wound healing constitute significant limitations to proper implant osseointegration. These abnormalities create a suboptimal biological environment, impeding the classical integration process between biomaterial and bone, especially when compounded by the microarchitectural and craniofacial morphological alterations characteristic of acromegaly. The next Section 3.4 situates these endocrine and inflammatory dynamics within the broader clinical context, including common comorbidities that may further modulate peri-implant healing and stability.

### 3.4. Pathophysiological Mechanisms and Comorbidities in Relation to Implant Integration

The systemic consequences of acromegaly for bone metabolism and soft tissues are well documented; however, their direct translation into implant therapy outcomes remains insufficiently supported by clinical and experimental data. In patients with acromegaly, trabecular and cortical microarchitectural alterations and an increased risk of fractures persist even after achieving biochemical control of the GH/IGF-1 axis, indicating long-term impairment of bone quality that may be relevant for primary and secondary implant stability [9].

Comorbidities frequently observed in patients with acromegaly, such as type 2 diabetes mellitus and cardiovascular disease, may additionally influence the outcomes of implant therapy. Clinical data directly addressing this population are very limited; however, observations from studies in the general population suggest a possible impact of these factors on the osseointegration process.

In a systematic review and meta-analysis, Chrcanovic et al. found that diabetic patients exhibited slightly greater marginal bone loss compared with healthy individuals, although implant survival rates did not differ significantly [18]. Similarly, Oates et al. observed that poor glycemic control appears to increase the risk of delayed healing and reduced implant stability, although the quality of evidence remains limited [19].

A more recent meta-analysis by Shang et al. showed that hyperglycemia may be associated with increased bleeding on probing, deeper peri-implant pockets, and a higher risk of implant failure [20].

Cardiovascular diseases, which are common in patients with acromegaly, may also play a role. In a case–control study, Wang et al. demonstrated that patients with cardiovascular disease had a significantly higher risk of moderate and severe peri-implantitis compared with controls [21]. Similar conclusions were drawn in a systematic review by Chu et al., which indicated a possible association between cardiovascular disease and an increased prevalence of peri-implantitis, although the authors emphasized the limited quality of available evidence [22].

In light of the above, it may be assumed that the coexistence of metabolic and cardiovascular disorders may increase the risk of implant-related complications in patients with acromegaly, although further studies are necessary to confirm this.

Regulation of osseointegration is multifactorial and involves not only GH and IGF-1 but also other hormones (e.g., parathyroid hormone), inflammatory mediators, and angiogenesis. Literature reviews indicate bidirectional interactions between the endocrine system and peri-implant tissue healing: GH and IGF-1 may promote early bone-to-implant contact (BIC) and bone remodeling in the initial healing phase, although this effect is strongly dependent on endocrine balance and local inflammatory status, factors that are often disrupted in active acromegaly [17]. As illustrated earlier in Figure 1, the balance between osteogenic and osteoclastic pathways is central to these interactions, with additional endocrine and inflammatory mediators further modulating the process

Preclinical data further suggest that targeted hormonal modulation may improve osseointegration parameters. In a meta-analysis of 18 animal studies, parathyroid hormone (PTH) supplementation significantly increased new bone formation and BIC compared with controls, supporting its potential to enhance implant integration under conditions of compromised bone biology [23]. Although these studies were not performed directly in acromegaly, they mechanistically correspond to the hormonal disturbances and bone remodeling changes described in these patients, suggesting potential therapeutic pathways that require clinical validation.

Direct clinical evidence in acromegaly remains very limited. A single case report documented successful implant therapy in a patient with acromegaly with a four-year follow-up period, indicating technical feasibility and functional maintenance when carefully planned and coordinated in an interdisciplinary setting; nevertheless, such isolated evidence does not allow generalization [5].

**Figure 2:**
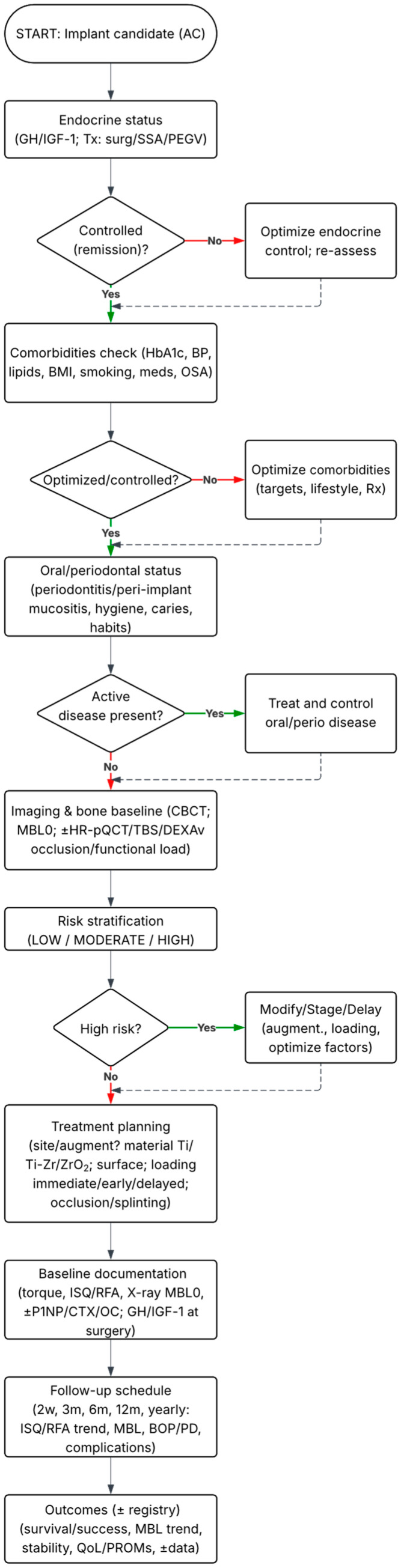
Proposed clinical decision pathway for implant therapy in patients with acromegaly. Decision nodes (diamonds) indicate clinical checkpoints (e.g., active vs. controlled disease, comorbidity optimization). Action boxes (rectangles) indicate recommended interventions (e.g., optimize endocrine therapy, treat periodontal disease, and modify surgical protocol). Follow-up and outcome documentation ensure long-term monitoring and research standardization. Abbreviations: GH = growth hormone; IGF-1 = insulin-like growth factor 1; CBCT = cone beam computed tomography; HR-pQCT = high-resolution peripheral quantitative computed tomography; TBS = trabecular bone score; ISQ/RFA = implant stability quotient/resonance frequency analysis; MBL = marginal bone loss; PROMs = patient-reported outcome measures.

In clinical practice, the profile of comorbidities frequent in acromegaly, including type 2 diabetes, hypertension, and dyslipidemia, appears to play a key role, as hyperglycemia, microangiopathy, and chronic inflammation may predispose to delayed healing, impaired immune function, and increased susceptibility to peri-implant infections. Although no studies have directly examined these interactions in acromegaly cohorts, evidence from diabetic populations consistently suggests a higher risk of suboptimal osseointegration and prolonged healing, particularly in poorly controlled glycemia. Therefore, assessment and optimization of comorbidities before implant placement, together with strict metabolic control, should be considered integral elements of clinical management (see Figure 2).

In summary, translating the pathophysiological mechanisms of acromegaly into implant outcomes requires structured evidence from prospective studies, including (i) standardized assessment of disease activity (GH, IGF-1), bone remodeling markers, and microarchitectural parameters; (ii) careful evaluation and documentation of comorbidities and their management; (iii) objective integration outcomes (primary/secondary stability, BIC from imaging or indirect measures, and marginal bone loss); and (iv) long-term clinical follow-up. Until such data are available, individualized treatment, preferably in states of hormonal remission and metabolic control, along with consideration of biomaterial strategies supporting healing in compromised bone beds, appears reasonable [9,17]. Acromegaly may be regarded as a “model disease” since it accumulates mechanisms potentially relevant for implant integration: excess GH/IGF-1, chronic low-grade inflammation, enhanced oxidative stress, and persistent alterations in trabecular and cortical microarchitecture [1,9,24]. Similar pathophysiological pathways are observed in more common conditions: in osteoporosis, impaired bone quality and remodeling may affect peri-implant outcomes [25]; in diabetes, hyperglycemia has been associated with increased bleeding on probing, greater peri-implant bone loss, and possibly higher failure risk [20]; and in metabolic syndrome, chronic inflammation and oxidative stress appear to contribute to peri-implantitis [26]. Consequently, insights from acromegaly—such as the need to optimize metabolic and inflammatory control before implantation and the potential value of antioxidant or immunomodulatory implant surfaces—appear to be translatable to patients with osteoporosis, diabetes, or metabolic syndrome, although this requires confirmation in prospective studies. This concept is consistent with the framework presented in Figure 1, emphasizing the convergence of hormonal, inflammatory, and oxidative mechanisms affecting bone–implant integration.

In patients with acromegaly, chronic exposure to excess growth hormone and IGF-1 alters bone metabolism and remodeling, leading to cortical thickening, disrupted trabecular organization, and increased bone turnover. These structural and biochemical changes may affect primary implant stability, osseointegration, and long-term bone–implant interface integrity. Although available evidence suggests that implant therapy can be successfully performed in acromegalic patients, the outcomes may depend on disease control, bone quality, and individualized surgical planning. Further prospective studies are needed to clarify implant survival rates, peri-implant tissue responses, and the influence of hormonal normalization on bone healing dynamics.

These pathophysiological and clinical determinants frame the material considerations addressed in Section 4, including standard and emerging biofunctional implant surfaces tailored to compromised bone homeostasis.

## 4. Characteristics of Biomaterials Used in Dental Implantology

In dental implantology, the selection of an appropriate implant material plays a pivotal role in ensuring durable osseointegration, biomechanical stability, and satisfactory esthetic outcomes. This raises the important question of whether traditionally employed solutions are sufficient in patients with acromegaly.

### 4.1. Standard Biomaterials

Titanium (Ti) and its alloys have represented the primary materials in dental implantology for several decades, owing to their unique combination of mechanical, biological, and chemical properties. Titanium demonstrates excellent biocompatibility and resistance to corrosion, which is attributed to the spontaneous formation of a superficial passive layer of TiO_2_. This layer is chemically inert and tightly adherent to the substrate, thereby substantially limiting the risk of immunological reactions and material degradation in the biological environment [27].

From a mechanical perspective, titanium is characterized by high tensile strength, a favorable elastic modulus, and strong fatigue resistance, which together provide long-term biomechanical stability of dental implants [28]. In addition, surface characteristics such as roughness and surface energy play a crucial role during the early phases of osseointegration. These properties promote osteoblast adhesion and support angiogenesis, both of which are essential for proper healing of peri-implant tissues [29].

In response to the demand for implants with enhanced mechanical performance, titanium alloys incorporating zirconium (Ti-Zr) were developed. These alloys—typically containing 13–17% zirconium, as in the commercially available Roxolid (Ti-15Zr)—exhibit greater hardness and a comparable elastic modulus to pure titanium. They also demonstrate improved resistance to cyclic loading, resulting in superior mechanical durability under long-term functional conditions [30]. Some studies further suggest that Ti-Zr alloys may provide enhanced corrosion resistance while maintaining high biocompatibility; however, the optimal alloy composition and definitive superiority over pure titanium remain to be conclusively established [31]. Preclinical and in vitro studies indicate that Ti-Zr alloys possess increased mechanical strength compared with pure titanium, enabling the use of thinner implants with greater load-bearing capacity [32].

Zirconia ceramics (ZrO_2_) have emerged as an alternative to metallic implants in dental implantology. They are particularly valued for their esthetics (white coloration), biocompatibility, and high corrosion resistance. Zirconia implants are especially indicated in patients with metal hypersensitivity and in esthetically demanding regions. Certain studies suggest that zirconia surfaces may exhibit lower bacterial adhesion compared with metallic surfaces, potentially reducing the risk of peri-implantitis, although further clinical validation is required [33]. Unlike metallic implants, zirconia does not cause discoloration of surrounding soft tissues and, due to its reduced bacterial affinity, may offer an additional advantage in minimizing peri-implant disease.

Standard implant surface modification techniques include methods that increase surface roughness, such as sandblasting combined with acid etching (SLA), anodization, and deposition of bioactive coatings containing calcium phosphate or hydroxyapatite. These procedures enhance bone-to-implant contact and osteoblast adhesion, thereby accelerating and improving the quality of osseointegration [34].

Overall, standard biomaterials, such as titanium, its alloys, zirconia ceramics, and surface-modified implants, constitute a robust and well-documented foundation of modern dental implantology. Their favorable mechanical, biological, and esthetic properties translate into high clinical success rates under typical conditions. However, in settings of impaired bone homeostasis, as in acromegaly, their use may necessitate individualized treatment strategies or supplementation with next-generation technologies.

In summary, conventional implant materials such as titanium, Ti–Zr alloys, and zirconia ceramics remain the clinical gold standard due to their excellent biomechanical strength, corrosion resistance, and biocompatibility. Surface modifications, including SLA and calcium-phosphate coatings, further enhance bone-to-implant contact and early osseointegration. However, under conditions of altered bone remodeling and chronic inflammation—such as in acromegaly—their long-term performance may be influenced by oxidative stress and impaired tissue regeneration. Further studies are warranted to evaluate whether additional surface functionalization or bioactive coatings could improve implant integration in these metabolically compromised environments.

### 4.2. Contemporary Advances in Dental Implantology

Current developments in dental implantology focus on the design of implants with bioactive surface coatings that not only promote osseointegration but also actively participate in modulating inflammation, angiogenesis, and the regeneration of both hard and soft tissues. Particular interest has been directed toward coatings functionalized with biomimetic peptides, growth factors, including bone morphogenetic proteins (BMPs), and agents that inhibit bacterial adhesion and biofilm development. The goal of such modifications is to simultaneously enhance bone integration and reduce the risk of bacterial colonization.

As demonstrated in a systematic review and meta-analysis by López-Valverde et al. (2022), titanium implant surface modifications incorporating bioactive molecules, particularly BMP-2, were shown in animal models to increase bone-to-implant contact (BIC) during short-term observation periods [35]. However, the authors emphasized considerable methodological heterogeneity among the included studies, as well as the limited generalizability of the findings, highlighting the need for further experiments with longer follow-up periods and improved standardization [35].

Evidence from preclinical in vitro studies and selected in vivo models suggests that implant coatings enriched with bioactive, biomimetic peptides—especially those containing the RGD (arginine–glycine–aspartic acid) sequence—may enhance osteoblast adhesion, proliferation, and differentiation. These mechanisms could positively influence the early stages of osseointegration. Nonetheless, most available data are derived from experimental studies, which limits the direct translation of these results into clinical practice [36,37].

In recent years, there has been increasing interest in implant coatings based on antimicrobial peptides and other compounds designed to reduce bacterial adhesion and biofilm formation on titanium surfaces. These strategies aim to mitigate the risk of peri-implant infections, including peri-implantitis, which remains one of the leading causes of implant failure. As reported by Akshaya et al. (2022), the effectiveness of such approaches depends on achieving a delicate balance between antimicrobial activity and the biocompatibility and osteogenic potential of the biomaterial surface [38]. The authors stressed that many proposed coatings remain in the preclinical phase, and their in vivo efficacy requires further validation [38].

Another promising direction involves the development of smart coatings capable of responding to local changes in the biological environment, such as pH variations, inflammatory cytokines, oxidative stress, or temperature shifts. These coatings can deliver active agents in a controlled manner, adapting their effects to the dynamic conditions at the implantation site. As emphasized by Joshi et al. (2023), such systems may be designed using environmental sensors, including light-responsive, thermosensitive, or pH-dependent matrices, creating opportunities for personalized treatment in complex clinical scenarios [39].

In dental implantology, self-adaptive antibacterial coatings represent an innovative strategy. These coatings respond to pH changes in the peri-implant microenvironment by releasing antibacterial agents under acidic conditions typical of inflammation, while simultaneously supporting osseointegration. The effectiveness of such approaches has been demonstrated in both in vitro and in vivo studies using titanium implants modified with pH-responsive coatings containing chitosan and cerium [40].

Particularly promising results have been reported for bioactive coatings inspired by natural structures. In the study by Zhou et al. (2025), a DOPA-P1@P2 coating based on mussel-inspired adhesive peptides was developed, demonstrating anti-inflammatory, angiogenic, and osteogenic properties [41]. In a preclinical rat model, this coating significantly accelerated bone regeneration, with a 207% increase in bone volume (BV/TV) and an over 1400% increase in bone-to-implant contact (BIC) compared with a TiO_2_ control group. These findings underscore the therapeutic potential of functional peptide-based coatings in implantology [41].

Although no implants have yet been designed specifically for patients with acromegaly, the biomaterials literature highlights emerging concepts of adaptive materials, such as porous composites and biomimetic surfaces, which may better interact with altered bone microarchitecture. Currently being explored primarily in orthopedics and metabolic bone disorders, these strategies provide a potential foundation for the future development of implants tailored to patients with endocrine-related skeletal abnormalities [42]. These adaptive surface technologies may also have relevance in systemic disorders such as acromegaly, where oxidative and inflammatory stress alter osseointegration dynamics (see Section 5).

Bioactive coatings incorporating peptides and growth factors, smart coatings responsive to local biological signals, and advanced adaptive materials represent new directions in dental implantology. Although these technologies remain largely experimental, their potential to enhance osseointegration—particularly in challenging pathological conditions such as acromegaly—warrants special attention and further investigation.

In summary, emerging biomaterial strategies—including bioactive coatings with peptides, growth factors, and antimicrobial agents, as well as smart, stimuli-responsive surfaces—represent a promising evolution of dental implant technology. These materials aim not only to promote osseointegration but also to modulate inflammation and resist bacterial colonization, offering potential advantages in compromised biological environments. However, the current evidence base remains largely preclinical, with substantial methodological heterogeneity and limited long-term data. Translation of these approaches to clinical practice, particularly in patients with systemic bone disorders such as acromegaly, will require well-designed, prospective studies to confirm their safety, durability, and true therapeutic benefit.

These material trends and biological mechanisms are further discussed in Section 5 in the context of altered bone homeostasis in acromegaly.

## 5. Interaction of Biomaterials with Bone in Acromegaly

### 5.1. Effects of GH/IGF-1 Excess on Implant Osseointegration: Preclinical and Clinical Data

In the context of acromegaly, characterized by chronically elevated levels of GH and IGF-1, it is crucial to evaluate whether modern implant biomaterials can effectively integrate with bone under conditions of disrupted endocrine homeostasis. Chronic hormonal stimulation may influence all stages of osseointegration, from the initial inflammatory response, through bone remodeling, to the final stabilization of the implant (see Table 3 for study synthesis).

Preclinical animal studies have demonstrated that, under controlled conditions, both GH and IGF-1 may partially support bone regeneration and biomaterial integration. In Stenport et al. (2001), systemic administration of human growth hormone (0.3 U/kg/day via subcutaneous pumps) in rabbits improved early implant stability, as measured by resonance frequency analysis (RFA) at 2 and 8 weeks [43]. No significant differences were observed in removal torque, dual-energy X-ray absorptiometry (DEXA), or histomorphometric analysis at 8 weeks. Antibody development occurred after 4 weeks, potentially limiting the duration of the anabolic effect. These results suggest that GH may enhance early implant stability but has no sustained effect on bone regeneration [43].

In the study by Abreu et al. (2015), the effects of local application of rhGH on the osseointegration of titanium implants in rabbits were evaluated [45]. After 3 and 6 weeks, the rhGH-treated group showed significantly greater BIC compared with controls. However, by 12 weeks, these differences had equalized, suggesting that GH may enhance early integration but does not substantially influence later phases of bone remodeling. Quantitative assessment of bone mineralization was not performed [45].

Tresguerres et al. (2002) investigated the effects of local rhGH administration in an osteoporotic rabbit model induced via bilateral ovariectomy [44]. Titanium implants were placed in the distal femur, and outcomes were compared between rhGH-treated and control groups. After 8 weeks, BIC was significantly higher in the GH group, along with improved histological organization of newly formed bone tissue. However, mineralization and trabecular bone microarchitecture were not evaluated. These results suggest that local GH administration may support osseointegration under conditions of impaired bone metabolism [44].

Conversely, in the study by López-Quiles et al. (2019), the effects of local IGF-1 application on the osseointegration of titanium implants in healthy rabbits were assessed [46]. After 8 weeks, BIC was significantly lower in the IGF-1 group compared with controls (37.4% vs. 46.3%, *p* = 0.032). Bone volume and mineralization were not evaluated. These findings suggest that local IGF-1 administration in healthy bone does not support osseointegration and may even impair it [46].

A summary of preclinical studies investigating the effects of GH/IGF-1 on implant osseointegration is presented in Table 3.

Despite the predominance of positive findings from preclinical research, clinical evidence remains extremely limited. To date, only a single case report has been published describing a patient with untreated acromegaly who underwent full implant prosthetic rehabilitation with a follow-up period of four years. Despite active GH excess and elevated IGF-1 levels, the titanium implants demonstrated stable clinical and radiological outcomes throughout the observation period. No radiographic signs of implant loss of osseointegration or peri-implant bone loss were observed; however, the authors emphasized the necessity of strict occlusal control, meticulous assessment of bone structure, and potentially wider safety margins when planning implant procedures [5].

Although large-scale clinical studies are lacking, existing in vivo experiments suggest that chronic GH and IGF-1 excess does not necessarily impair the osseointegration process. Under controlled conditions, it may even support bone regeneration by enhancing osteoblast activity and accelerating new bone formation, as demonstrated in animal models. Nevertheless, the biological environment of a patient with active acromegaly requires thorough evaluation—both in terms of local anatomical conditions and systemic endocrine control. The success of implant therapy in this patient group appears to depend more on the optimization of treatment planning tailored to the individual biology of the patient than on the inherent properties of the biomaterial itself.

In summary, acromegaly represents a unique biological model for investigating the interaction between systemic endocrine disturbances and local bone–implant dynamics. Chronic GH/IGF-1 excess induces structural and metabolic alterations in bone—characterized by increased turnover, trabecular disorganization, and cortical porosity—that compromise mechanical anchorage. Concurrently, dysregulated inflammatory and angiogenic responses can affect both early healing and long-term osseointegration. The use of biomaterials with bioactive or adaptive surface properties, as outlined in Section 4, may mitigate these unfavorable biological conditions by promoting osteogenesis and modulating inflammation.

Section 5.2 discusses how specific implant surface modifications and material technologies can address these pathophysiological challenges and improve osseointegration outcomes under acromegalic conditions.

### 5.2. Implant Biomaterials Under Acromegalic Conditions

In the context of chronic hyperactivity of the GH/IGF-1 axis, as observed in acromegaly, significant alterations occur within the bone and mucosal microenvironment. These include increased oxidative stress, modulation of inflammatory pathways, and elevated expression of pro-inflammatory cytokines. Such factors may negatively affect the adhesive properties, biocompatibility, and durability of implant biomaterials, thereby hindering the osseointegration process, particularly under conditions of impaired bone remodeling.

Acromegaly leads to a chronic increase in GH and IGF-1 levels, resulting not only in accelerated bone remodeling but also in qualitative changes, such as deterioration of trabecular structure (reduced trabecular number, increased trabecular separation) and increased cortical porosity, as well as oxidative stress and chronic inflammation within bone tissues [1]. Under such conditions, standard titanium implant surfaces may not be sufficiently resilient; delayed responses of osteogenic cells, an increased risk of marginal bone loss, and weaker BIC (bone-to-implant contact) may occur.

Studies on titanium surface modification indicate that the application of nitrogen plasma treatment (non-thermal nitrogen plasma) to commercially pure titanium surfaces improves hydrophilicity, reduces carbon contamination, introduces nitrogen-containing functional groups, and promotes cell adhesion and proliferation, as well as in vivo osseointegration, without significant changes in surface roughness or morphology [47]. Such improvement in surface properties may be particularly beneficial in patients with acromegaly, in whom metabolic alterations and oxidative stress limit the natural capacity for regeneration and mineralization.

Furthermore, a literature review on the generation of reactive oxygen species (ROS) on titanium implant surfaces and on surface-modification strategies to enhance antioxidant properties provides a range of evidence that surfaces designed to reduce oxidative stress exhibit better biocompatibility and favor improved osseointegration [48]. Organic and inorganic coatings, composite materials containing antioxidants, and polymer coatings that respond to ROS or include free-radical-scavenging groups were correlated in these studies with reduced oxidative damage, increased osteoblast proliferation, and improved osteogenic differentiation.

Another example of a strategy to improve osseointegration is the removal of hydrocarbon contaminants from the implant surface through surface cleaning/gas plasma. In the study by Hyungyu Lee and Hyun Jeong Jeon et al., “Improvement of osseointegration efficacy of titanium implant through plasma surface treatment,” was reported. Dielectric barrier discharge (DBD) plasma treatment was applied to SLA implants (sand-blasted, large grit, acid-etched), which made it possible to reduce carbon contamination by ~60% and to increase adhesion, proliferation, and differentiation of osteogenic cells under in vitro conditions [49].

These examples show that there are biomaterials and surface-modification technologies that address some of the key metabolic problems of acromegaly: excess ROS, alterations in bone structure, collagen disturbances, and slower mineralization. In the context of acromegaly, implant surface design must take the following into account:

- Hydrophilicity and surfaces containing functional groups that interact with the protein layer;
- The ability to reduce organic carbon contaminants that may inhibit protein and cell adhesion;
- Surface features resistant to oxidative stress (e.g., antioxidant coatings), nanotube type, or textured structures, and chemical groups that neutralize ROS;
- Coating stability under variable conditions: in patients with active acromegaly, hormonal and metabolic conditions may be unstable, which requires the coating to be durable and resistant.

Electrochemical and microscopic studies have demonstrated that reduced pH destabilizes the passive titanium dioxide (TiO_2_) layer, which plays a critical protective role against corrosion. In acidic environments, the corrosion resistance of both pure titanium and titanium alloys decreases, leading to increased metal ion release and surface degradation of the implant [50]. Within the oral environment, low pH may result from bacterial biofilm, fluoride-containing mouth rinses, or an acidogenic diet. These factors contribute to the degradation of the protective oxide layer on titanium surfaces, thereby increasing the risk of implant corrosion [51].

Chronic inflammation promotes increased production of reactive oxygen species (ROS), which further accelerates titanium implant corrosion. ROS can damage the protective TiO_2_ layer, contributing to material degradation and exerting cytotoxic effects on osteogenic cells, even in implants with oxidized coatings [52].

Excess ROS also activate macrophages and osteoclasts, resulting in elevated secretion of pro-inflammatory cytokines such as IL-1β and TNF-α. This intensifies local inflammation and increases the risk of peri-implantitis, ultimately leading to bone degradation. Moreover, titanium implants may release nano- and microscale particles through adhesive and mechanical wear processes. These particles can activate inflammasomes and amplify local inflammatory responses [48].

Corrosion studies under simulated inflammatory conditions demonstrated that both reduced pH and the presence of ROS destabilize the protective TiO_2_ layer, causing pitting corrosion in the material. These microenvironmental changes increase the risk of titanium particle and ion release into surrounding tissues, potentially inducing chronic inflammation and compromising long-term implant stability [53].

Furthermore, as highlighted by Souza et al. (2020), factors such as bacterial biofilm, an acidogenic diet, and fluoride-based oral rinses may lower pH and promote implant surface corrosion [51]. The released particles, depending on their size and composition, may trigger inflammatory reactions that contribute to peri-implantitis [51].

Comparative studies of titanium implants have also demonstrated that prosthetic abutments are particularly susceptible to corrosion when exposed to aggressive environmental factors, such as strongly acidic pH (e.g., 2.5). Under these conditions, the passive TiO_2_ layer becomes destabilized, leading to impaired electrochemical properties and surface structural alterations, thereby reducing corrosion resistance [54].

The altered conditions present in patients with acromegaly, including low pH, heightened oxidative stress, and chronic inflammation, may thus contribute to degradation of implant materials, destabilization of the passive oxide layer, and release of metallic particles and ions. Such an environment may compromise implant surface integrity and increase the risk of osseointegration failure.

In summary, titanium and zirconia remain the most clinically validated implant materials, yet their performance in acromegaly may be compromised by oxidative stress, inflammatory activation, and altered bone remodeling. Emerging biomaterial strategies—such as antioxidant coatings, plasma-treated surfaces, and bioactive nanostructures—show promise in mitigating these challenges, although clinical validation is lacking.

Section 5.3 reviews available clinical and retrospective data to assess whether these experimental insights translate into successful implant therapy in patients with acromegaly.

### 5.3. Clinical Cases and Retrospective Reports of Treatment

The number of clinical reports on dental implantology in patients with acromegaly remains limited; however, the few available publications provide important clinical observations that allow for assessment of the actual feasibility of prosthetic rehabilitation in this patient group.

The most significant of these is the case of a 59-year-old male patient with active acromegaly who underwent full dental implantation. Over a four-year follow-up period, the implants maintained clinical and radiological stability despite persistent GH and IGF-1 excess. The authors emphasized that the success of the procedure was achieved through meticulous surgical planning, consideration of bone remodeling, careful occlusal control, and close collaboration with the endocrinologists [5].

Another case, concerning rehabilitation with removable dentures in a patient with acromegaly, highlighted the importance of achieving hormonal disease stabilization prior to initiating dental treatment. Lack of GH control led to irreversible skeletal changes that prevented effective denture adaptation, thereby limiting therapeutic options [55].

Analyses of prosthetic cases, including complete dental rehabilitation with removable prostheses, confirm that altered craniofacial anatomy—particularly chronic changes such as mandibular prognathism—requires an individualized approach and often necessitates delaying treatment until hormonal remission is achieved [56].

Although long-term controlled implantology studies in acromegaly are lacking, these retrospective clinical observations and case reports provide several key conclusions. First, implants can achieve successful osseointegration even under conditions of chronic GH/IGF-1 excess, provided the patient is in a stable treatment phase and under strict endocrine control. Second, prosthetic management in such patients requires an interdisciplinary approach, precise surgical planning, long-term endocrine stabilization, and continuous assessment of changes in maxillomandibular anatomy and biomechanics.

For ease of comparison of the available data on bone microarchitecture and implant treatment outcomes in patients with acromegaly, a synthetic summary is provided in Table 4. The table outlines the range of applied methods (MDCT, HR-pQCT, and TBS), the main imaging results, and the limited clinical data available on implants.

Clinical data on implantology in patients with acromegaly remain extremely limited. To date, only a single case report has documented titanium implants functioning successfully over a four-year follow-up period [5]. Other publications have described only prosthetic rehabilitation, with no prospective or cohort studies assessing implant survival in this patient group. Therefore, it should be emphasized that most of the conclusions in this study are based on preclinical data and literature concerning general metabolic disorders and biomaterials.

Most available data are case reports or small case series (the lowest level of clinical evidence), with no controlled trials or long-term follow-up. While such reports are valuable for generating hypotheses, they provide only limited guidance for clinical practice. This reflects the rarity of acromegaly, but also highlights the need for prospective cohort studies. The proposed clinical decision pathway for implant therapy in patients with acromegaly—integrating endocrine evaluation, systemic risk control, oral and periodontal health, bone quality assessment, and individualized treatment planning—is illustrated in Figure 2.

The mechanisms observed in acromegaly—excessive bone remodeling, oxidative stress, and dysregulated inflammation—are also characteristic of more prevalent metabolic disorders, including diabetes mellitus, osteoporosis, and metabolic syndrome. Consequently, therapeutic concepts explored in acromegaly, such as antioxidant, peptide-functionalized, and smart biomaterial coatings, may hold broader translational relevance. These interdisciplinary strategies could inform the design of next-generation implant surfaces optimized for osseointegration in metabolically compromised bone.

Acromegaly exemplifies the convergence of systemic hormonal dysregulation and local bone pathology in determining implant outcomes. Achieving endocrine control and selecting biomaterials tailored to the patient’s metabolic profile are crucial for predictable integration. Although current evidence remains preliminary and heterogeneous, it provides a sound scientific basis for the future development of bioadaptive implant surfaces with potential applications across a range of metabolic bone disorders.

## 6. Design and Material Implications for the Future

### 6.1. Development Directions of Implant Materials Under Altered Bone Homeostasis

The growing understanding of the effects of chronic GH/IGF-1 axis stimulation on the bone microenvironment in acromegaly underscores the need for next-generation implant surfaces capable of modulating inflammatory responses, reacting to biological signals, and supporting regeneration under conditions of impaired bone homeostasis.

One of the major challenges remains the control of peri-implant inflammatory responses, particularly regarding cytokines such as IL-1β and TNF-α, which contribute to peri-implantitis and disrupt osseointegration. Increasing attention has been directed toward immunomodulatory coatings capable of limiting inflammatory cell activation and supporting tissue repair through modulation of the cytokine milieu. As reviewed by Dong et al. (2022), advanced biomaterial strategies—including the incorporation of anti-inflammatory agents or immunomodulatory molecules directly into the implant surface—may effectively reduce inflammation and enhance implant integration [57].

In dental implantology, so-called smart biomaterials, which are engineered materials capable of dynamically responding to local environmental stimuli such as pH, enzymatic activity, or inflammatory mediators, have attracted growing interest. Their adaptability enables precise, signal-dependent release of bioactive agents, potentially supporting hard tissue regeneration while mitigating adverse immunological or bacterial processes at the implant site.

For example, in the design of materials for caries management, He et al. (2023) discussed pH-responsive drug delivery systems engineered to release antimicrobial agents exclusively under acidic biofilm conditions, thereby providing selective therapeutic action while sparing healthy tissues [58]. Similarly, Han et al. (2023) reviewed stimuli-responsive nanotechnologies for titanium implant surface modifications designed to react to pH, enzymatic cues, or external stimuli that demonstrate both antibacterial and osteopromotive properties [59]. Furthermore, Zhang et al. (2022) developed a porous, shape-memory self-adaptive scaffold that autonomously conforms to bone defects, exhibits antibacterial properties, and promotes bone regeneration in animal models, which is an applied example of adaptive smart biomaterials for implantology [60].

Particularly promising are smart polymeric biomaterials that respond to local physicochemical stimuli such as temperature, pH, or specific bacterial enzymes. These polymers can be applied as implant coatings or three-dimensional scaffolds, enabling signal-dependent, controlled release of antimicrobial or osteogenic factors. Preclinical studies with photo-reactive and thermosensitive scaffolds have demonstrated accelerated wound healing and bone regeneration [61].

Another innovative strategy involves bioinspired biomaterials capable of mechanosensory activation in response to local mechanical loading. For instance, piezoelectric scaffolds generate localized electric charges under mechanical stress, thereby enhancing osteoblast differentiation and extracellular matrix mineralization. Such properties may promote osseointegration in unstable microenvironments or in the presence of structural deficits. Although still largely preclinical, this technology represents a promising approach in bone tissue engineering and next-generation implant design [62].

In the context of acromegaly, where profound alterations in bone metabolism are observed, the application of smart biomaterials responsive to local biological conditions may represent a breakthrough in bone regeneration. Owing to their adaptive properties, these materials can buffer increased biomechanical loads, support osteogenesis, and modulate inflammatory responses at the implant–bone interface, thereby significantly improving implant therapy outcomes in patients with compromised bone homeostasis.

### 6.2. Opportunities for Implant Personalization

Advances in three-dimensional (3D) printing and additive manufacturing (AM) technologies have opened new clinical perspectives in dental implant design, enabling customization to the patient’s individual anatomical and biological needs. In implantology, particularly in the setting of metabolic disorders such as acromegaly, individualization of both implant geometry and material properties becomes crucial to optimize integration with remodeled bone structure and to account for GH/IGF-1 axis disturbances.

Additive manufacturing techniques, such as selective laser melting (SLM) and direct metal laser sintering (DMLS), have revolutionized the fabrication of medical implants. These approaches allow for the production of highly complex geometries with precisely controlled porosity, thereby promoting superior integration with bone through enhanced osseointegration and vascularization. They are increasingly applied in the creation of personalized implants and tissue-supporting structures, such as custom-designed titanium meshes for bone augmentation. By integrating patient-specific imaging data (CT, CBCT) into digital modeling systems, such structures can be optimally adapted to irregular anatomical conditions, which are critical for complex reconstructions, e.g., after trauma, oncological resections, or advanced alveolar ridge atrophy. The use of titanium alloys such as Ti-6Al-4V ensures optimal mechanical and biocompatible properties. Notably, recent progress in combining AM with tissue engineering techniques may enable the fabrication of cell-seeded or growth factor-coated structures [63].

In the context of dental implant personalization, 3D printing allows the fabrication of anatomically accurate structures tailored to the morphology of the patient’s maxilla or mandible. This approach reduces the extent of surgical preparation, enhances primary implant stability, and optimizes bone–implant contact surface. Integration of CT imaging and intraoral scanning, processed via CAD/CAM systems, enables highly precise surgical planning and the fabrication of individualized components, such as surgical guides, temporary prostheses, and custom geometry implants [64].

In acromegaly, where bone undergoes remodeling and biomechanical alterations, porous implant structures represent a particularly promising solution for improving biomaterial–bone integration. Porosity reduces the phenomenon of stress shielding, in which excessive load transfer through the implant occurs at the expense of bone loading, leading to bone resorption and compromised implant stability. Controlled porosity further supports osteogenic cell adhesion and vascular ingrowth, thereby enhancing osseointegration [65,66].

Additive manufacturing enables precise control over porosity parameters, including pore size, graded porosity, and internal architecture, which are critical for matching implant strength and elasticity to bone mechanics and counteracting stress shielding. For example, lattice-type Ti-6Al-4V structures manufactured layer by layer enable the design of implants with optimized, graded porosity tailored to the patient’s biomechanical profile, minimizing adverse bone-loading effects [67]. Additionally, AM technologies allow for surface biofunctionalization; for instance, deposition of hydroxyapatite, strontium, or silver in a single manufacturing process, thereby simultaneously promoting osseointegration and imparting antibacterial properties [68].

In acromegaly, where increased bone mass, altered geometry, and remodeling abnormalities are common, porous, personalized implants fabricated via AM may provide biomechanical compatibility by locally reducing elastic modulus in areas requiring greater bone load distribution, while simultaneously enhancing tissue integration.

Moreover, 3D printing has facilitated the development of composite implants, such as combinations of biodegradable polymers with hydroxyapatite or nanocarbons. These materials can be engineered for the gradual release of osteogenic factors and support of bone remodeling, which may be particularly advantageous in pathological metabolic conditions [69]. Thus, the convergence of 3D printing technologies and biocomposites holds promise for creating implants that are simultaneously anatomically customized, mechanically compatible, and biologically active. Although further research is warranted, these technologies may significantly enhance the safety and efficacy of implant therapy in high-risk populations, particularly in patients with acromegaly.

### 6.3. Potential Directions for Future Research

Future development of dental implantology in the context of acromegaly should focus on several key areas of investigation. Experimental models employing transgenic animals with GH or IGF-1 overexpression could provide valuable insights into the effects of chronic hormonal stimulation on osseointegration and bone regeneration. Such models would enable the identification of the biological mechanisms underlying potential disturbances in biomaterial integration and support the development of tailored therapeutic strategies. However, to date, no such models have been established, representing a significant gap in the literature and highlighting the need for further preclinical studies.

In parallel, prospective clinical studies involving patients with acromegaly undergoing implant therapy are of paramount importance. Optimal study design should include patients with varying hormonal profiles and degrees of endocrine control, while monitoring long-term outcomes of osseointegration and prosthetic stability. The realization of such studies would require close interdisciplinary collaboration among implantologists, endocrinologists, bone biologists, and biomaterials engineers, as well as the establishment of sufficiently large clinical registries. Only such an approach would allow a comprehensive understanding of which hormonal, metabolic, and biomechanical factors determine the success of implant therapy in this unique patient population.

In vitro models represent another essential research tool for analyzing the impact of metabolic disturbances on the integration of implant biomaterials. Under laboratory conditions, osteoblast cultures or bone tissue samples can be stimulated with factors characteristic of acromegaly, such as elevated GH and IGF-1 levels, increased concentrations of pro-inflammatory cytokines (IL-6, TNF-α), and oxidative stress. These experiments allow for precise evaluation of how pathological microenvironmental conditions affect adhesion, proliferation, and mineralization of osteogenic cells on biomaterial surfaces. They are also employed to test bioactive and adaptive implant coatings designed to counteract the remodeling disturbances typical of acromegalic patients. For instance, Locatelli and Bianchi (2014) demonstrated that GH increases IL-6 production by osteoblast-like cells, potentially promoting bone resorption via remodeling mechanisms [70]. Conversely, according to observations by Tahimic et al. (2013), IGF-1 enhances survival, proliferation, osteoblast differentiation, and bone matrix synthesis—both in vitro and in vivo—confirming its key anabolic role in bone homeostasis [71].

Alongside experimental research, computational modeling (in silico) is gaining increasing importance as a rapid and cost-effective tool for simulating biomaterial degradation, bone remodeling, and the formation of the bone–implant interface. For example, Pohl et al. (2025) developed a mathematical model based on differential equations describing the degradation of magnesium implants and osseointegration [72]. The model incorporated parameters such as relative bone volume and ultrastructure of surrounding tissue, achieving a mean prediction error for bone volume fraction (BVF) of less than 6% [72]. Similarly, Gandía et al. (2025) conducted a systematic review of current computational models used to assess titanium implant osseointegration, evaluating finite element analysis (FEA), mechanobiological approaches, and reaction–diffusion models [73]. The authors emphasized the need for integrated validation protocols and improved incorporation of biological components into biomechanical models [73].

In light of the limitations identified in the current literature and the real clinical needs of patients with acromegaly, we propose a five-component research agenda as a set of priorities for validation, encompassing preclinical models, in vitro studies, translational/clinical studies, the development of personalized implants and 3D printing technologies, and computational modeling and predictive biomarkers.

First, it may be valuable to consider the development of animal models of acromegaly (e.g., with GH or IGF-1 overexpression), which could help determine whether chronic stimulation of the GH/IGF-1 axis influences implant osseointegration in a manner distinct from standard models. To date, studies on acromegaly have mainly focused on the hormonal level and systemic bone parameters, without direct assessment of implant osseointegration in this context, which appears to represent a notable research gap. In such models, analyses should include bone-to-implant contact (BIC), marginal bone loss, micro-CT, and biomechanical parameters (removal torque/pull-out strength), along with concurrent mapping of inflammation and oxidative stress. At the same time, the translational and ethical limitations of transgenic models must be emphasized, as well as the fact that they do not fully capture clinical complexity (comorbidities, pharmacotherapy, and genetic heterogeneity).

Second, in vitro studies could be designed to more faithfully replicate the microenvironment of acromegaly: high GH/IGF-1 levels, pro-inflammatory cytokines (e.g., IL-6, TNF-α), and oxidative stress/acidic pH. Under these conditions, osteoblasts, osteoclasts, precursor cells, and macrophages should be tested for proliferation, differentiation (e.g., Runx2, BMP-2, and OCN), angiogenesis, and immune responses, both on standard materials (Ti, Ti-Zr, and ZrO_2_) and on bioactive/”smart” coatings. A recent review suggests that hormones such as GH and IGF-1 may modulate osseointegration both directly and indirectly (through immune or vascular pathways) [17]. Moreover, findings from 3D-printed structures (PLA/HA/GNP) have shown improved osteoblast adhesion, osteogenic differentiation, and reduced inflammatory responses under in vitro conditions [74]. These results appear promising but require further verification, particularly in vivo.

Third, translational and clinical studies represent another key area, especially prospective cohorts of patients with acromegaly undergoing implant therapy. Such studies should be stratified based on GH/IGF-1 axis activity (active disease vs. remission), disease duration, age, comorbidity profile, and bone metabolism status (e.g., P1NP, CTX, and osteocalcin). It is important to note that the prognostic value of these biomarkers for osseointegration in acromegaly has not been established and should be analyzed in an exploratory manner. Clinical outcomes (implant stability, marginal bone loss, function, and biological complications) should be reported at least annually over long-term follow-up (see Figure 2 for the monitoring schedule). Current clinical data are extremely limited (isolated case reports of successful implant therapy) [5]. In contrast, preclinical interventions involving growth hormone have shown beneficial effects on BIC and new bone formation in animal models, findings that have also been confirmed in a meta-analysis [75] and in a rabbit study [45]. Translation of these results into clinical practice for acromegaly may require further well-controlled prospective trials.

Fourth, the development of personalized implants and materials with controlled porosity and gradient structures appears to be a promising direction, particularly in the context of altered bone geometry and loading in acromegaly. Preclinical data in biomaterials engineering (e.g., biomimetic mineralization of porous bioglass scaffolds; hydroxyapatite coatings on porous polymeric implants such as PEKK) suggest improvements in osteogenic markers and cell adhesion [76,77]. However, no clinical data in acromegaly are currently available; early “first-in-human” studies under strict safety oversight may be warranted.

Finally, computational modeling and predictive biomarkers should support the selection of materials and therapeutic strategies. Finite element analysis (FEA) models should incorporate the microarchitectural alterations observed in acromegaly (e.g., Tb.N, Tb.Sp, Ct.Po, and Ct.Th) and functional loading, and then be validated against clinical data (MBL trajectories, ISQ/RFA trends) and where available, structural indices (e.g., TBS/HR-pQCT). A meta-analysis of animal studies suggests that GH interventions may increase BIC [75], which may serve as a rationale for considering the design of clinical studies with predictive endpoints in humans.

The studies cited in this review are characterized by several limitations, including small sample sizes, heterogeneity of study designs, and the predominance of descriptive or preclinical data. Most clinical reports involve isolated cases or short follow-up periods, with limited biochemical or histomorphometric assessment. Experimental studies, while informative, often employ simplified in vitro conditions that only partially reproduce the biological environment observed in acromegaly. Consequently, the overall level of evidence remains low, and the conclusions of this review should be interpreted with caution until validated by larger, controlled, and long-term studies.

In summary, integrating five elements—(1) preclinical models replicating GH/IGF-1 excess, (2) in vitro studies under “disease-like” conditions, (3) prospective cohorts with clear endocrine stratification and MBL/ISQ reporting, (4) personalized/3D implants with controlled porosity and bioactive coatings, and (5) FEA modeling validated with biomarkers—may provide conceptual frameworks and a potential roadmap for future studies. Such a plan could enable the transition from hypotheses and preclinical data to validated clinical recommendations, while potentially limiting the risk of overinterpretation and inconsistent results

These directions underscore the growing need for interdisciplinary collaboration among endocrinologists, material scientists, and clinicians to develop bioadaptive implants optimized for hormonally altered bone.

## 7. Conclusions

1. Osseointegration of dental implants in patients with acromegaly is feasible, as confirmed by both preclinical data (animal models, GH/IGF-1 application) and isolated clinical observations. However, successful treatment requires individualized assessment of bone status and strict metabolic control in continuous collaboration with the treating endocrinologist.
2. The anatomical and biological conditions observed in patients with acromegaly represent a significant challenge for classic osseointegration. Chronic GH/IGF-1 stimulation, impaired bone remodeling, persistent inflammation, and altered maxillomandibular morphology adversely affect implant stability and functionality.
3. Standard biomaterials, such as titanium and its alloys, may undergo degradation under conditions associated with acromegaly, including increased oxidative stress and chronic inflammation. Therefore, material solutions with higher chemical resistance and enhanced biofunctionality are required.
4. Next-generation implants may demonstrate superior adaptability to altered conditions through the use of bioactive, immunomodulatory, and stimuli-responsive surfaces (e.g., responsive to pH changes, cytokine presence, or oxidative stress).
5. Acromegaly may serve as a model disease that integrates mechanisms relevant for implantology, such as altered bone microarchitecture, chronic inflammation, oxidative stress, and metabolic imbalance. Insights gained from this rare condition may be translatable to more common disorders, including osteoporosis, diabetes, and metabolic syndrome.
6. Personalization of implants using additive manufacturing technologies is becoming a key element of treatment. Three-dimensional printing, the design of controlled-porosity structures, and the application of composite biomaterials enable the creation of solutions tailored to a patient’s individual metabolic and anatomical alterations.
7. Future research should include animal models with GH/IGF-1 overexpression, in vitro experiments simulating acromegalic conditions, and prospective clinical trials. These data are essential for defining the actual design requirements of biomaterials in this specific patient population.
8. While acromegaly cannot be considered an absolute contraindication to implant therapy, the condition requires cautious, individualized treatment planning, close interdisciplinary coordination, and careful selection of biomaterials suited to the altered biological environment.

## Figures and Tables

**Table 1:** Bone microarchitectural changes in acromegaly.

Parameter	Healthy Individuals	Active Acromegaly	Acromegaly in Remission	Source	Study Type/Evidence Level	Main Limitations
Trabecular number (Tb.N)	Normal	Decreased	Persistently decreased	Duan 2021 [1]; Kuker 2023 [3]	Clinical cohort study/Level III	Small samples; cross-sectional design; limited fracture data
Trabecular separation (Tb.Sp)	Low	Increased	Persistently increased	Duan 2021 [1]; Kuker 2023 [3]	Clinical cohort study/Level III	HR-pQCT only distal radius/tibia; no histology
Cortical porosity	Low	Elevated	Elevated	Duan 2021 [1]; Madeira 2013 [2]; Kuker 2023 [3]	Observational clinical study/Level III	Different imaging methods; no longitudinal follow-up
aBMD	Normal	Normal or ↑	Frequently ↑ despite structural deficits	Madeira 2013 [2]; Giustina 2023 [9]	Mixed clinical + review data/Level III–V	DXA overestimates strength; lacks volumetric context
TBS (trabecular bone score)	High	Low	Low	Giustina 2023 [9]	Narrative review/Expert consensus (Level V)	Expert synthesis; not primary data

Evidence levels: III—observational clinical; IV—case series; V—expert opinion or review. All studies referenced in this table correspond to verified publications: Duan et al., 2021 [1] (J Clin Endocrinol Metab); Madeira et al., 2013 [2] (Eur J Endocrinol); Kuker et al., 2023 [3] (Bone); Giustina et al., 2023 [9] (Endocrinol Metab (Seoul).

**Table 2:** Morphological alterations of the maxilla and mandible in acromegaly.

Feature	Prevalence	Implantological Implications	Sources	Study Type/Evidence Level	Main Limitations
Mandibular prognathism	20–22% of patients	Malocclusion; may require orthognathic surgery	De Stefani 2022 [4]; Gosau 2009 [10]; Pereira 2024 [11]	Case reports and narrative review/Level IV–V	Small sample size; descriptive only; no functional/biomechanical assessment
Macroglossia	54–58% of patients	Prosthetic adaptation difficulties; risk of occlusal overload	De Stefani 2022 [4]; StatPearls 2025 [12]	Narrative review/Level V	No quantitative data; absence of implant-specific correlations
Diastemas	40–43% of patients	Impacts esthetics and prosthetic planning	De Stefani 2022 [4]	Narrative review/Level V	Based on cross-sectional review; no clinical outcomes
Condylar process overgrowth	Reported in case studies; no prevalence statistics	Favors Class III malocclusion; potential indication for orthognathic surgery	Gosau 2009 [10]	Case report/Level IV	Single-patient data; no longitudinal follow-up or implant outcome data

Evidence levels: IV—case series or case report; V—expert opinion or narrative review. All studies referenced in this table correspond to verified publications: De Stefani 2022 [4] (J Craniofac Surg); Gosau 2009 [10] (Oral Surg Oral Med Oral Pathol Oral Radiol Endod); Pereira 2024 [11] (Front Dent Med); and StatPearls 2025 [12] (StatPearls Publishing).

**Table 3:** Effects of GH/IGF-1 on osseointegration: preclinical studies.

Author (Year)	Model/Condition	Intervention (Dose, Route)	Observation Period	Main Osseointegration Outcome	Notes	Limitations
Stenport 2001 [43]	Rabbit; Ti implants in tibia	hGH systemically 0.3 U/kg/day via subcutaneous pumps	2 and 8 weeks	↑ stability by RFA at 2 and 8 weeks; no differences in removal torque, DEXA, or histomorphometry at 8 weeks	Antibodies detected after 4 weeks; transient early effect	Limited duration due to immunogenicity; small N; short follow-up
Tresguerres 2002 [44]	Rabbit osteoporosis (OVX + low-calcium diet); Ti implants in tibia	rhGH locally 4 IU to osteotomy (lyophilized powder)	2 weeks	↑ peri-cortical and trans-cortical reaction and osteoid mineralization at 14 days	Early anabolic response observed histologically	Single early time-point; histology only; no BIC/biomechanics reported.
Abreu 2015 [45]	Rabbit; Ti implants in tibia	rhGH locally 1 IU (0.3 mg) to osteotomy on the implant	3, 6, and 12 weeks	↑ BIC and pull-out strength at 3 and 6 weeks; no differences at 12 weeks	Acceleration of the early healing phase	Small N; short follow-up; limited quantitative BIC/mineralization data
López-Quiles 2019 [46]	Rabbit (healthy vs. osteoporotic OVX + low-calcium diet); Ti implants in tibia	IGF-1 locally 4 μg to osteotomy	8 weeks	↓ BIC vs. controls in healthy rabbits (37.4% vs. 46.3%, *p* = 0.032); no benefit in osteoporotic model	Dose/time-dependent response; limited bone formation	Single dose; no bone-volume or mineralization quantification

RFA = resonance frequency analysis; DEXA = dual-energy X-ray absorptiometry; BIC = bone-to-implant contact; OVX = ovariectomy. ↑—increase; ↓—decrease.

**Table 4:** Summary of imaging and clinical data in acromegaly in context of implantology.

Author, Year	Method	Population	Findings (Microarchitecture/Implants)	Notes	Evidence Level	Main Limitations
Duan 2021 [1]	HR-pQCT	55 patients with acromegaly (active + remission) vs. healthy controls	↓ trabecular number, ↑ separation, ↑ cortical porosity, normal/increased BMD	Persistent changes even after remission	III (observational clinical)	Cross-sectional design; no implant data; limited sample; no fracture or functional correlation
Giustina 2023 [9]	TBS	Cohort of patients with acromegaly	Low TBS despite normal BMD	Fracture risk is underestimated using DXA	V (narrative review/expert synthesis)	Not original data; no direct implant outcomes; limited methodology detail
Schiller 2020 [6]	Case report	1 patient with acromegaly	Successful bone augmentation	No metabolic data available	IV (case report)	Single patient; no biochemical/endocrine data; unknown disease activity
Kernen and Bidra 2019 [5]	Case report	1 patient with acromegaly	Stable implants over 4 years	Only reported case of implantation in acromegaly	IV (case report)	Isolated case; lacks control; limited generalizability

Evidence levels: III—observational clinical study; IV—case series or case report; V—expert opinion or narrative review. ↑—increase; ↓—decrease.

## Data Availability

No new data were created or analyzed in this study. Data sharing is not applicable to this article.
